# Mechanism‐informed machine learning for individualized tacrolimus dose adjustment in the early post‐kidney transplant period

**DOI:** 10.1002/bcp.70448

**Published:** 2026-01-13

**Authors:** Hui Yu, Zihan Qin, Logan S. Smith, Jeong M. Park, Hao‐Jie Zhu

**Affiliations:** ^1^ Department of Pharmaceutical Sciences University of Michigan Ann Arbor MI 48109 USA; ^2^ Department of Clinical Pharmacy University of Michigan Ann Arbor MI 48109 USA

**Keywords:** kidney transplantation, machine learning, pharmacokinetics, precision dosing, tacrolimus

## Abstract

**Aim:**

Tacrolimus dosing in the early post‐kidney transplant period is challenging due to a narrow therapeutic index and substantial interindividual pharmacokinetic (PK) variability. This study aimed to develop and validate mechanism‐informed machine learning (ML) models to support individualized tacrolimus dosing during this critical period.

**Methods:**

A total of 4311 tacrolimus trough concentrations (C_trough_) within 7 days post‐transplant were obtained from 1624 kidney transplant recipients. Two ML models, Gated Recurrent Unit (GRU) and eXtreme Gradient Boosting (XGBoost), were developed to predict C_trough_ and recommend doses to achieve target levels. Both models incorporated PK principles based on linear pharmacokinetics. Model performance was compared to a traditional Bayesian population PK (PopPK) model and purely data‐driven ML models via internal cross‐validation and external validation.

**Results:**

The mechanism‐informed GRU model outperformed the Bayesian PopPK model in both internal validation (MSE = 7.81 *vs*. 9.27 ng^2^/mL^2^, R^2^ = 0.537 *vs*. 0.450) and external validation (MSE = 6.09 *vs*. 8.96 ng^2^/mL^2^, R^2^ = 0.397 *vs*. 0.211). The mechanism‐informed XGBoost model performed comparably to the GRU model. The incorporation of PK principles enhanced model interpretability and generalizability without reducing accuracy. When clinically administered doses, determined by conventional therapeutic drug monitoring, fell within the GRU model's recommended range, subsequent C_trough_ reached the therapeutic target (8–12 ng/mL) in 51.3% of cases, compared to 37.0% overall (p < 0.01).

**Conclusion:**

Mechanism‐informed ML models offer a robust and interpretable approach for individualized tacrolimus dosing, with the potential to improve therapeutic target attainment by enabling accurate dose adjustments in the early post‐transplant period.

**What is already known about this subject**

- Tacrolimus is a cornerstone immunosuppressant in kidney transplantation, but it has a narrow therapeutic index and exhibits substantial interindividual pharmacokinetic variability.
- Current dosing strategies rely on therapeutic drug monitoring (TDM) and empirical dose adjustments, which are often suboptimal during the early post‐transplant period.
- Model‐Informed Precision Dosing (MIPD) has been proposed to enhance individualized tacrolimus dosing and improve target attainment.

**What this study adds**

- Mechanism‐informed machine learning (ML) models, including Gated Recurrent Unit (GRU) and eXtreme Gradient Boosting (XGBoost), demonstrated superior accuracy in predicting tacrolimus trough concentrations compared to traditional Bayesian population pharmacokinetic models.
- Incorporating pharmacokinetic principles into ML models improved model interpretability and generalizability without compromising predictive performance.
- A publicly accessible tool based on the mechanism‐informed GRU model was developed to illustrate its potential for individualized tacrolimus dose adjustment in clinical settings.

## 1 INTRODUCTION

Tacrolimus is a first‐line calcineurin inhibitor used as part of combination immunosuppressive therapy in kidney transplantation, playing a critical role in preventing acute rejection and improving allograft survival.^1^ However, optimizing tacrolimus therapy remains a significant clinical challenge due to its narrow therapeutic index and substantial inter‐ and intra‐patient pharmacokinetic (PK) variability.^1^, ^2^ Failure to achieve and maintain target exposure can lead to detrimental consequences.^3^, ^4^ Several studies have shown that delays in reaching therapeutic levels during the early post‐transplant period are associated with an increased risk of acute rejection,^5^, ^6^, ^7^ whereas supratherapeutic levels can result in serious adverse effects such as nephrotoxicity, neurotoxicity and increased infection susceptibility.^4^

To guide individualized tacrolimus dosing, therapeutic drug monitoring (TDM) is routinely employed in clinical practice.^8^ While the area under the concentration‐time curve (AUC) is considered a better marker of tacrolimus exposure, trough concentration (C_trough_) is currently used in most transplant centres due to its practicality and correlation with AUC.^9^, ^10^ Initial dosing is typically weight‐based, with subsequent dose adjustments empirically guided by TDM results. However, during the early post‐transplant period, C_trough_ is often measured before steady‐state is achieved due to frequent dose changes.^11^ Additionally, this period is characterized by dynamic physiological changes, such as fluctuations in haematocrit, serum albumin, renal and hepatic function and fluid status, that contribute to the high PK variability during this phase and complicate dosing adjustments.^12^, ^13^ Currently, no standardized protocol exists to guide dose adjustment during this critical period; physicians often rely on personal experience and clinical judgement.^14^ Studies have reported that fewer than 40% of patients reach target tacrolimus C_trough_ at first steady‐state,^15^ and it may take up to 2–3 weeks to reach the target exposure.^15^, ^16^

Model‐informed precision dosing (MIPD) has been proposed to improve individualized dosing by integrating mathematical models with patient‐specific information.^14^, ^17^ Population PK (PopPK) modelling is the most established MIPD approach and has been applied to guide both a priori dosing based on baseline covariates and a posteriori adjustments using observed drug concentrations in kidney transplant recipients.^16^, ^18^ Evidence from retrospective and prospective studies demonstrates that PopPK‐guided dosing improves the achievement of therapeutic targets.^11^, ^19^ However, the performance of PopPK models can vary substantially across patient populations and clinical settings.^20^ In patients with rapidly changing physiological conditions, such as those in the early post‐kidney transplant period, PopPK models may fail to adequately capture the complexity and dynamic nature of patient physiology.

In recent years, machine learning (ML) approaches have shown promise in enhancing the precision of tacrolimus dosing.^21^, ^22^ ML models excel at utilizing temporal data, such as prior dosing history, serial TDM measurements and evolving clinical covariates, to learn complex dose–concentration relationships and improve prediction accuracy. Studies have reported that ML models often outperform traditional PK models in predicting tacrolimus exposure and individualizing dosing.^21^, ^23^, ^24^ Tang et al. were among the first to apply ML models to predict stable tacrolimus doses in renal transplant recipients.^25^ Subsequent studies demonstrated that approaches such as ExtraTreesRegressor and TabNet yield strong performance in concentration and dose prediction.^26^, ^27^ Long short‐term memory (LSTM) networks have also been effective in predicting tacrolimus levels in liver and lung transplant recipients.^21^, ^28^ Additional studies have explored ML‐based dosing in kidney transplant patients during the perioperative period.^22^, ^29^ However, ML models are often criticized as ‘black boxes’, lacking the interpretability and physiological grounding necessary for clinical adoption. Moreover, unlike mechanistic models such as PopPK, purely data‐driven ML models may not generalize well to clinical scenarios beyond their training data.

This study aims to develop and validate mechanism‐informed ML models to support a posteriori individualization of tacrolimus dose based on TDM data during the early post‐kidney transplant period. To enhance physiological interpretability, we incorporated PK principles based on linear pharmacokinetics with first‐order absorption and elimination. Two mechanism‐informed ML models were developed: a Gated Recurrent Unit (GRU) neural network and an eXtreme Gradient Boosting (XGBoost) model. Model performance was evaluated through internal cross‐validation and external validation using an independent cohort and compared with a conventional Bayesian PopPK model as well as purely data‐driven ML models. Our goal is to establish a modelling framework that integrates PK principles with ML to support individualized and timely tacrolimus dose optimization.

## 2 METHODS

### 2.1 Study design

This retrospective cohort study included adult kidney transplant recipients who received tacrolimus‐based immunosuppressive therapy at the University of Michigan Hospital between January 2001 and January 2025. The study was approved by the Institutional Review Board at the University of Michigan.

#### 2.1.1 Patient population

Eligible patients were ≥18 years of age at the time of kidney transplantation and received oral, immediate‐release tacrolimus (Prograf or generic equivalents) twice daily as part of their standard immunosuppressive regimen. Patients were included if they had complete records of tacrolimus dosing and at least two C_trough_ measurements obtained within the first 7 days post‐transplant. C_trough_ was defined as whole blood tacrolimus levels measured immediately prior to the next scheduled dose.

Exclusion criteria were as follows: (1) multi‐organ transplantation or re‐transplantation; (2) age <18 years; (3) incomplete or missing tacrolimus dosing information; (4) use of non‐oral or extended‐release tacrolimus formulations.

#### 2.1.2 Immunosuppression and laboratory analysis

The standard immunosuppressive regimen at the University of Michigan Hospital for adult kidney transplant recipients consists of tacrolimus, mycophenolate mofetil and a steroid taper. Tacrolimus therapy is initiated within 24 h post‐transplant at an initial dose of 0.075 mg/kg, administered orally every 12 h. Subsequent dose adjustments are guided by TDM using whole blood C_trough_.

Tacrolimus C_trough_ measurements were performed at Michigan Medicine Laboratories using a validated liquid chromatography–tandem mass spectrometry (LC–MS/MS) assay, with a lower limit of quantification (LLOQ) of 1.0 ng/mL. Additional methodological details are available at https://mlabs.umich.edu/tests/tacrolimus.

### 2.2 Data collection and preprocessing

Tacrolimus administration times, doses and C_trough_ values were extracted from the electronic medical records for the first 7 days following kidney transplantation.

The following variables were collected for each patient:

Demographics: age, sex and race.

Anthropometrics: weight, height, body mass index (BMI) and body surface area (BSA).

Concomitant medications: use of known Cytochrome P450 3A4/3A5 (CYP3A4/5) inhibitors and inducers, based on U.S. Food and Drug Administration (FDA) guidance.^30^

Lifestyle factors: documented history of smoking and alcohol use.

Laboratory values: haematocrit, albumin, alanine aminotransferase (ALT), aspartate aminotransferase (AST) and serum creatinine.

Race was categorized as Caucasian, African American or Other. Smoking and alcohol use were encoded as binary variables, with patients classified as ‘Yes’ if there was any documented history of use (current or former), and ‘No’ otherwise. The use of CYP3A4/5 inhibitors or inducers was recorded if administered within three days prior to each TDM measurement and encoded as binary variables. For each dose administration and C_trough_ measurement, the laboratory value closest in time was selected. Missing lab values were imputed using linear interpolation between adjacent time points. If no adjacent values were available, the nearest available value was carried forward or backward.

CYP3A4/5 genotype data were available for only 23.2% of patients in the University of Michigan Hospital dataset and were unavailable in the external validation dataset, MIMIC‐IV. Therefore, genotype information was excluded from the final analysis.

### 2.3 Population pharmacokinetic modelling

A Bayesian PopPK modelling approach was implemented using non‐linear mixed‐effects modelling in Monolix (version 2024R1; Lixoft, Antony, France). Given the sparse nature of the dataset (C_trough_ only), the structural model described by Francke et al.^31^ was adopted.

This model, which was originally developed on rich PK data from 1180 kidney transplant recipients, is a two‐compartment model with first‐order absorption. In our analysis, all structural parameters (apparent volumes of distribution, intercompartmental clearance and absorption‐related parameters) were fixed to the published estimates. Apparent clearance (CL/F) was re‐estimated using our study data. Because pharmacogenetic data (e.g., CYP3A5 genotype) were unavailable, genotype‐related covariate effects in the original model were removed. A new covariate model for CL/F was developed via stepwise forward selection and backward elimination using available demographic and laboratory covariates in our dataset. Inter‐individual variability was estimated in CL/F using an exponential model, and a proportional error model was used to describe the residual unexplained variability.

To evaluate the predictive performance of the PopPK model, a posteriori forecasting simulation was performed. Population parameters were first estimated from the training set and then fixed. For each patient in the test set, empirical Bayes estimates (EBEs) of individual PK parameters were updated sequentially using prior C_trough_ measurements (C_trough,1_ … C_trough,n‐1_) to predict the next concentration (C_trough,n_).

### 2.4 Machine learning model development

Two ML models were developed: GRU, a type of Recurrent Neural Network (RNN) well‐suited for modelling temporal dependencies in longitudinal datasets, and XGBoost, a robust ensemble method known for high performance in structured clinical data.^32^

Both models incorporated PK principles based on linear pharmacokinetics with first‐order absorption and elimination, which have been shown to adequately describe tacrolimus C_trough_ in kidney transplant recipients.^33^ The general equation for drug concentration at time t following multiple oral doses is:

(1) $$C\left(t\right)=\sum_{i=1}^{n}\frac{F\cdot k_{a}}{V_{d}\cdot \left(k_{a}-k_{e}\right)}D_{i}\cdot \left(e^{-k_{e}\left(t-t_{i}\right)}-e^{-k_{a}\left(t-t_{i}\right)}\right)$$

where D_i_ is the dose administered at time t_i_, k_a_ is the absorption rate constant, k_e_ is the elimination rate constant, V_d_ is the volume of distribution and F is the bioavailability.

Immediate‐release tacrolimus is rapidly absorbed, with peak concentrations occurring within 0.5–2 h after oral administration.^34^ By the time of trough measurement (approximately 12 h post‐administration), the absorption phase is largely complete. Therefore, when modelling only trough concentrations, the exponential term $e^{-k_{a}\left(t-t_{i}\right)}$ in Equation (1) becomes negligible with t − t_i_ ≈ 12 and k_a_ ≫ k_e_. Under this approximation, Equation (1) simplifies to:

(2) $$C\left(t\right)=\sum_{i=1}^{n}\alpha \cdot D_{i}\cdot e^{-k_{e}\left(t-t_{i}\right)}$$

where $\alpha =\frac{F\cdot k_{a}}{V_{d}\cdot \left(k_{a}-k_{e}\right)}\approx \frac{F}{V_{d}}$ is a biologically informed composite parameter that influences drug exposure.

**FIGURE 1:**
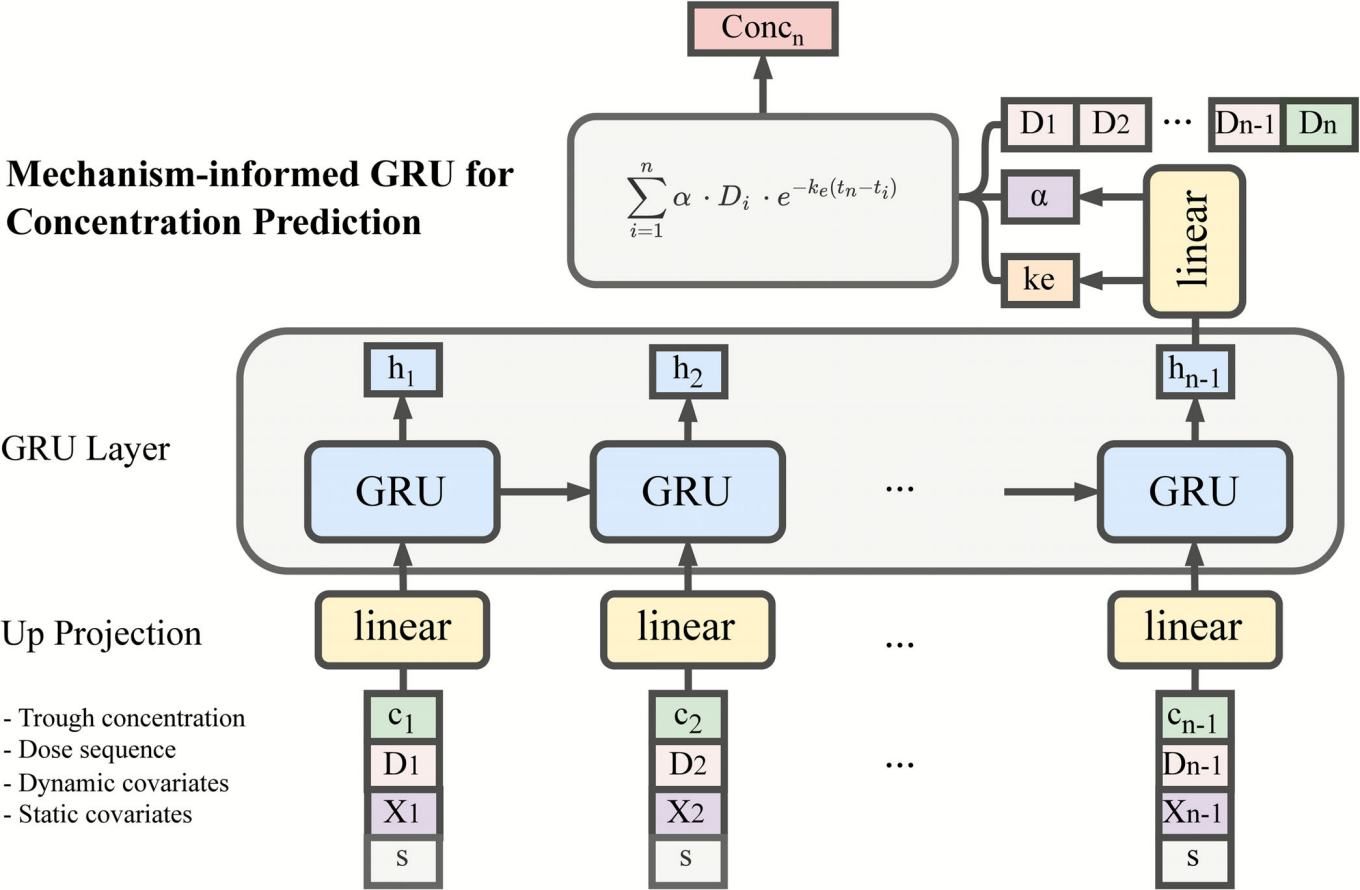
Structure of the mechanism‐informed machine learning model. The model consists of a single gated recurrent unit (GRU) layer that receives concatenated inputs comprising: (1) historical dose sequences (D_i_), (2) prior tacrolimus trough concentrations (c_i_) and (3) dynamic clinical covariates (X_i_). Static patient‐specific covariates (s) are concatenated with the sequential inputs at each time step. The GRU processes the sequential data, and the final hidden state (h_n − 1_) is passed through two parallel fully connected (dense) layers to estimate two parameters, α and k_e_, and predict the tacrolimus trough concentration.

For the GRU model, patient data were structured as sequential time‐series inputs to preserve temporal ordering. Each sequence represented a series of observations during the 7‐day post‐transplant period. To accommodate variable‐length patient histories, a sequence padding strategy was implemented. For each patient, observations were sorted chronologically. At each time point (excluding the first), a prediction window was constructed using all prior time points. These sequences were zero‐padded to match the length of the longest sequence within each batch. Padding lengths were tracked and masked during both training and inference to ensure that padding tokens did not introduce noise or bias into the model. The model architecture included a single GRU layer that received concatenated inputs composed of: (1) historical dose sequences, (2) prior tacrolimus C_trough_ and (3) time‐varying clinical features (e.g., haematocrit, albumin, creatinine). Static patient‐specific features (e.g., age, sex, race) were concatenated with the sequential inputs at each time step, allowing the model to contextualize temporal data with baseline characteristics. The GRU output at the final time step was passed through two parallel fully connected (dense) layers to estimate two parameters, α and k_e_. The estimated α and k_e_ were then utilized to calculate C_trough_ based on Equation (2). During training, k_e_ was constrained to a physiologically plausible range (0.017–0.17 h^−1^) based on literature values for adult kidney transplant recipients.^35^ No explicit constraint is imposed on α, allowing the model greater flexibility to capture inter‐individual variability in drug exposure. The model was trained using the Adam optimizer (learning rate = 0.001) for up to 30 epochs, with a loss function based on mean squared error (MSE) between predicted and observed C_trough_ values. The overall model architecture is illustrated in Figure 1.

In contrast to neural network‐based models, XGBoost, based on gradient‐boosted decision trees, cannot jointly optimize parameters α and k_e_ via loss‐based gradient descent. Instead, these parameters must be pre‐specified or estimated externally and incorporated as fixed inputs or engineered features. To adapt the PK constraints for XGBoost, k_e_ was set either to a fixed population average value or to an individual estimate obtained from a PopPK model. To capture longitudinal patterns, a set of time‐dependent lag features was constructed. These lag features included the remaining drug amount calculated as $\sum_{i=1}^{n}D_{i}\cdot e^{-k_{e}\left(t-t_{i}\right)}$, the observed C_trough_, an empirical estimate of α, computed as the ratio of C_trough_ to the remaining drug amount, as well as time‐varying covariates. All of these features were constructed using the two most recent TDM measurements prior to the target observation. Additional time features included time after transplantation, time after the first dose and time interval. We trained the model to predict α using constructed lag features, static patient‐specific features and time features. The predicted α was then combined with the estimated remaining drug amount to compute the predicted C_trough_ using Equation (2). The model was trained by minimizing the MSE between the predicted and observed C_trough_ values. To ensure unbiased hyperparameter tuning and robust performance estimation, a nested cross‐validation framework was employed. Within each outer training set, an inner four‐fold cross‐validation was conducted to tune hyperparameters. The best‐performing hyperparameters from the inner loop were used to train the model on the outer training set, which was subsequently evaluated on the corresponding test fold.

To compare the mechanism‐informed ML models with purely data‐driven approaches, we developed direct C_trough_ prediction models using both GRU and XGBoost, excluding PK principles. The purely data‐driven GRU model retained the same architecture and input structure as the mechanism‐informed version. However, instead of optimizing the parameters α and k_e_, the output at the final valid time step was directly passed through a dense layer to predict C_trough_. The optimal model weights were determined via grid search over a predefined hyperparameter space. For the purely data‐driven XGBoost model, the prior remaining drug amount and the prior empirical estimate of α were not included as input lag features​. Instead, the model used daily dose directly, along with the same other features as in the mechanism‐informed model. A nested cross‐validation framework was employed for hyperparameter tuning and performance evaluation.

All models were implemented in Python (v3.12.10), using PyTorch (v2.8.0) for the GRU‐based models and XGBoost (v3.0.2) for the XGBoost‐based models.

### 2.5 Model evaluation and comparison

All models were evaluated using five‐fold cross‐validation. The dataset was split into five folds using patient‐level stratified cross‐validation, ensuring that all data from a given patient were assigned to a single fold to prevent data leakage. In each fold, models were trained on four folds (training set) and evaluated on the remaining fold (test set). Model performance was averaged across the five folds to obtain unbiased estimates. Performance metrics included MSE, mean absolute error (MAE), and coefficient of determination (R^2^), mean percentage error (MPE) and root mean squared relative percentage error (RMSRPE).

### 2.6 Feature importance analysis

To assess the contribution of each input variable to model predictions, we performed feature importance analyses specific to each model type. For the GRU model, we used a permutation‐based approach in which each input feature was randomly shuffled across the test set, thereby disrupting its relationship with the target variable. The resulting increase in prediction error (measured by the change in MSE) was used to quantify the relative importance of each feature. A larger increase in MSE indicated a greater contribution of that feature to model performance. For the XGBoost model, feature importance was evaluated using gain‐based metrics, which quantify the average improvement in model performance (i.e., reduction in loss) contributed by a feature across all decision tree splits where it was used.

### 2.7 External validation

External validation was conducted using the MIMIC‐IV database, which includes de‐identified clinical data from patients admitted to Beth Israel Deaconess Medical Center (Boston, MA, USA) between 2008 and 2019. Adult patients who received tacrolimus and met the inclusion criteria outlined in the *Patient Population* section were selected. Relevant demographic, clinical and laboratory variables were extracted to align with the University of Michigan Hospital cohort and underwent the same data preprocessing procedures.

### 2.8 Dose recommendation strategy

To generate individualized tacrolimus dose recommendations, the mechanism‐informed GRU model estimates individual parameters (α and k_e_) based on longitudinal data, including prior dosing history, TDM results, laboratory results and demographic information. Using these parameters, the model forecasts future tacrolimus C_trough_ and applies optimization techniques to determine the dosing regimen required to achieve a specified target C_trough_. Doses are assumed to be administered at regular 12‐hour intervals and are rounded to the nearest 0.5 mg in accordance with clinical practice.

## 3 RESULTS

### 3.1 Cohort characteristics and tacrolimus dosing and concentrations

After applying the inclusion and exclusion criteria, 4311 tacrolimus measurements from 1624 patients were included in the analysis. The demographic and clinical characteristics of the study cohort are summarized in Table 1. The mean age of the study cohort was 51.9 years, and 62.7% of patients were male. The majority of patients were Caucasian (66.1%), followed by African American (23.7%).

**TABLE 1:** General characteristics of the study cohort and external validation cohort.

Characteristic	Study cohort (N = 1624)	External validation cohort (N = 164)
Age (years)	51.9 ± 13.8	53.6 ± 13.2
Gender	Male 1018 (62.7%)	Male 106 (64.6%)
	Female 606 (37.3%)	Female 58 (35.4%)
Race	Caucasian 1074 (66.1%)	Caucasian 82 (50.0%)**
	African American 385 (23.7%)	African American 42 (25.6%)
	Other 165 (10.2%)	Other 40 (24.4%)
Tobacco Use	No 901 (55.5%) Yes 723 (44.5%)	/
Alcohol Use	No 1066 (65.6%) Yes 558 (34.4%)	/
CYP3A Inhibitor Use	No 1292 (79.6%) Yes 332 (20.4%)	/
CYP3A Inducer Use	No 1539 (94.8%) Yes 85 (5.2%)	/
Height (cm)	171.9 ± 10.2	171.7 ± 10.8
Weight (kg)	87.4 ± 21.1	81.7 ± 16.9**
BMI (kg/m^2^)	29.5 ± 6.2	27.6 ± 4.7**
BSA (m^2^)	2.03 ± 0.28	1.97 ± 0.24
Serum Creatinine (mg/dL)	3.54 ± 2.61	4.35 ± 3.50
Haematocrit (%)	27.9 ± 4.3	26.5 ± 3.1**
Albumin (g/dL)	3.49 ± 0.36	4.15 ± 0.58**
ALT (U/L)	31.3 ± 49.9	21.4 ± 22.9**
AST (U/L)	32.6 ± 42.4	21.6 ± 13.1**
Pharmacokinetic data		
Dose (mg/day)	11.8 ± 3.6	10.1 ± 4.0**
Trough Level (ng/mL)	9.7 ± 4.0	6.7 ± 2.3**
Number of samples	4311	611
Number of samples per patient	2.7 ± 1.0	3.7 ± 1.2

*Note*: ** Indicates a statistically significant difference (p < 0.01) between the external validation cohort and the study cohort. P‐values were calculated using Welch's t‐test for continuous variables and the chi‐square test for categorical variables.

Abbreviations: ALT, alanine aminotransferase; AST, aspartate aminotransferase; BMI, body mass index; BSA, body surface area. Continuous variables are reported as mean ± standard deviation. Categorical variables are presented as counts (percentages).

As shown in Table 1 and Supplementary Figure S1, most daily tacrolimus doses ranged from 8 to 15 mg, with a mean of 11.8 mg. Doses exceeding 15 mg/day were less common, and only a few surpassed 20 mg/day. The majority of tacrolimus C_trough_ values fell between 5 and 15 ng/mL, with a mean of 9.7 ng/mL. However, some patients exhibited markedly high trough concentrations exceeding 30 ng/mL, particularly within the first 48 h after the initial dose. These observations highlight the substantial inter‐individual variability in tacrolimus PK during the early post‐transplant period.

The distribution of TDM measurements per patient in the study cohort and temporal changes in C_trough_ are illustrated in Supplementary Figures S2 and S3. Most patients (60.3%) had only two TDM measurements within the first 7 days post‐transplant. The maximum number of TDM measurements recorded for a single patient during this period was six, with an average of 2.7 measurements per patient. The temporal distribution of TDM measurements following tacrolimus initiation is presented in Supplementary Table S1. The highest measurement density was observed in the 24–48 h interval (n = 1572), followed by 48–72 h (n = 1370). The number of observations declined thereafter, with only 55 measurements recorded in the 144–168 h interval.

### 3.2 Comprehensive evaluation of prediction algorithms

We developed two mechanism‐informed ML models, GRU and XGBoost, to predict tacrolimus C_trough_. Model performance was compared with a Bayesian PopPK model and purely data‐driven ML models. Details of the PopPK model are summarized in Supplementary Tables S2, S3 and goodness‐of‐fit results are presented in Supplementary Figure S4. The comparative performance of various modelling approaches in predicting C_trough_ is summarized in Table 2.

**TABLE 2:** Performance of different models for tacrolimus trough concentration prediction in internal cross‐validation and external validation.

Model type	Model	MSE (ng^2^/mL^2^)	MAE (ng/mL)	R^2^	MPE	RMSRPE	P10	P20	P30
**Internal cross‐validation (N = 1624)**									
Mechanism‐informed ML	GRU	7.81	2.02	0.537	4.77%	30.9%	29.2%	55.5%	73.9%
	XGBoost	7.89	2.04	0.532	6.12%	31.9%	29.3%	55.1%	73.1%
Purely data‐driven ML	GRU	7.85	2.03	0.534	5.09%	31.6%	28.2%	54.8%	73.8%
	XGBoost	7.98	2.03	0.527	4.52%	30.8%	28.7%	55.6%	73.4%
PopPK	Bayesian forecasting	9.27	2.32	0.450	13.7%	39.6%	25.5%	48.5%	65.4%
**External validation (N = 164)**									
Mechanism‐informed ML	GRU	6.09	1.71	0.397	−3.22%	27.5%	34.9%	57.1%	74.7%
	XGBoost	5.95	1.69	0.411	−4.21%	26.4%	33.6%	57.5%	75.4%
Purely data‐driven ML	GRU	6.84	1.87	0.322	−8.68%	27.9%	28.2%	55.5%	74.9%
	XGBoost	7.27	1.96	0.279	−13.9%	27.7%	25.7%	48.1%	70.9%
PopPK	Bayesian forecasting	8.96	2.22	0.211	6.18%	38.8%	22.2%	46.3%	64.7%

Abbreviations: GRU, Gated Recurrent Unit; ML, Machine Learning; MAE, Mean Absolute Error; MPE, Mean Percentage Error; MSE, Mean Squared Error; P10, P20, P30: proportion of prediction error within ±10%, ±20%, ±30%, respectively; PopPK, Population Pharmacokinetics; R^2^, Coefficient of Determination; RMSRPE, Root Mean Squared Relative Percentage Error; XGBoost, eXtreme Gradient Boosting.

The mechanism‐informed GRU model consistently outperformed the Bayesian PopPK model across all metrics, achieving lower MSE (7.81 *vs*. 9.27 ng^2^/mL^2^), MAE (2.02 *vs*. 2.32 ng/mL) and higher R^2^ (0.537 *vs*. 0.450). It also demonstrated lower MPE (4.77% *vs*. 13.7%) and RMSRPE (30.9% *vs*. 39.6%) compared to the PopPK model, suggesting that the GRU model not only reduces absolute and relative prediction errors but also minimizes systematic bias. Furthermore, the GRU model yielded higher proportions of predictions within ±10%, ±20% and ±30% of observed C_trough_ values (P10: 29.2%, P20: 55.5%, P30: 73.9%) compared with the PopPK model (P10: 25.5%, P20: 48.5%, P30: 65.4%), highlighting its improved predictive accuracy and potential utility for individualized dose optimization.

The mechanism‐informed XGBoost model, implemented with a fixed population elimination rate constant (k_e_ = 0.0578 h^−1^), achieved comparable performance to the GRU model, with an MSE of 7.89 ng^2^/mL^2^, MAE of 2.04 ng/mL and R^2^ of 0.532. We also explored an integrated ML–PopPK approach by incorporating individualized k_e_ values estimated from prior TDM data. However, this approach resulted in reduced predictive accuracy (MSE of 9.45 ng^2^/mL^2^ and MAE of 2.25 ng/mL), likely due to the PopPK model's limited ability to accurately estimate individual parameters from sparse early‐phase data.

Additionally, we evaluated a purely data‐driven approach that directly predicted tacrolimus C_trough_ without incorporating PK principles. The purely data‐driven ML models showed comparable performance to their mechanism‐informed counterparts in the internal cross‐validation, with all models achieving an MSE < 8.0 ng^2^/mL^2^ and an R^2^ around 0.53. These results suggest that integrating PK principles does not compromise predictive accuracy and may offer added interpretability and clinical relevance.

### 3.3 External validation

To assess generalizability, all models were externally validated on a cohort from the MIMIC‐IV database, which included 164 kidney transplant patients who met the study's inclusion/exclusion criteria, with a total of 611 tacrolimus concentration measurements. Demographic and clinical characteristics of this external cohort are summarized in Table 1, and the distributions of tacrolimus doses and C_trough_ are presented in Supplementary Figure S5. Compared with the study cohort, the external cohort had a lower proportion of Caucasian patients and lower body weight, BMI, haematocrit and liver enzyme levels (ALT, AST). Notably, patients in the external cohort received lower daily tacrolimus doses (10.1 ± 4.0 *vs*. 11.8 ± 3.6 mg/day) and exhibited lower trough levels (6.7 ± 2.3 *vs*. 9.7 ± 4.0 ng/mL), which might be due to different protocols adopted by different transplant centres.

**FIGURE 2:**
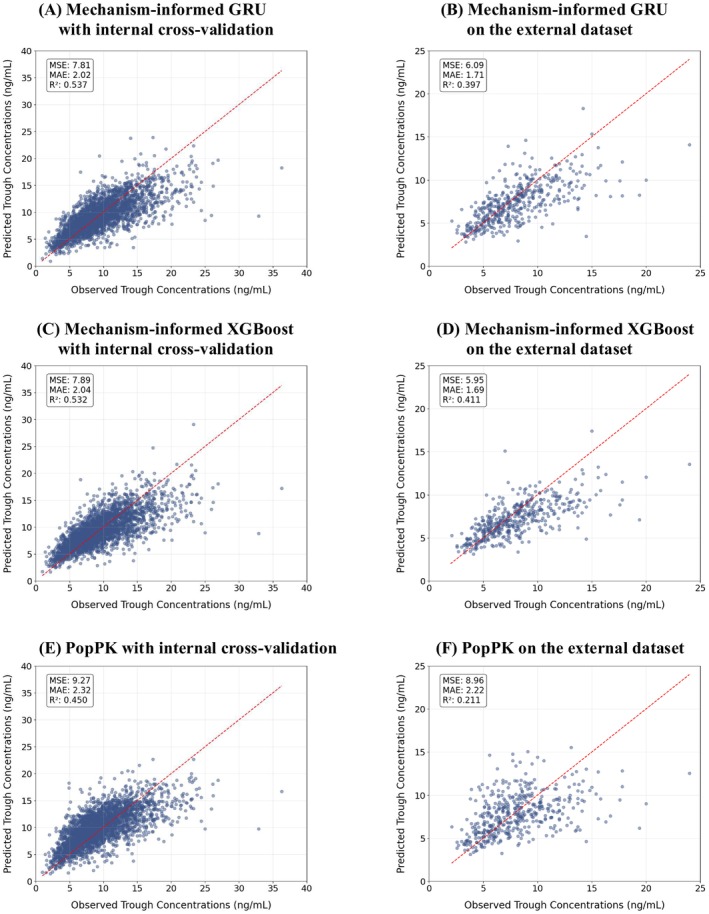
Predicted *vs.* observed tacrolimus trough concentrations across all cross‐validation folds and the external validation dataset for different models. (a) Mechanism‐informed GRU model with internal cross‐validation. (b) Mechanism‐informed GRU model on the external dataset. (c) Mechanism‐informed XGBoost model with internal cross‐validation. (d) Mechanism‐informed XGBoost model on the external dataset. (e) PopPK model with internal cross‐validation. (f) PopPK model on the external dataset. The red dashed line represents the line of identity.

The final models, trained exclusively on data from the University of Michigan Hospital, were applied to the external cohort without any retraining or fine‐tuning. As shown in Table 2 and Figure 2, both mechanism‐informed GRU and XGBoost models outperformed the PopPK model in the external validation (MSE 6.09 and 5.95 *vs*. 8.96 ng^2^/mL^2^; MAE 1.71 and 1.69 *vs*. 2.22 ng/mL). To further assess model robustness, we conducted a stratified analysis of GRU model performance by race in both cohorts. For the Caucasian subgroup, clinical accuracy (P30) remained stable between the study (73.4%) and external (74.8%) cohorts, though bias (MPE) shifted from overprediction (4.50%) to underprediction (−2.34%) and R^2^ decreased (0.51 to 0.37). Similarly, for the African American subgroup, P30 was comparable between cohorts (study: 73.4%; external: 71.0%), with a shift in bias from 5.79% to −3.88%. The observed shift in predictive bias and lower R^2^ are likely attributable to substantial physiological and protocol differences between the study and external cohorts.

Compared to the mechanism‐informed ML models, the purely data‐driven ML models that directly predicted C_trough_ without PK constraints demonstrated inferior performance on the external dataset. To investigate the cause of this discrepancy, we examined the dose–concentration relationships learned by the models. As shown in Supplementary Figure S6, the purely data‐driven XGBoost model exhibited an irregular, stepwise relationship between dose and predicted C_trough_, lacking the expected linear trend. Similarly, in the purely data‐driven GRU model, doubling the dose did not consistently result in a corresponding increase in predicted C_trough_. In contrast, both mechanism‐informed XGBoost and GRU models preserved a linear and proportional dose–concentration relationship, consistent with established pharmacological principles. This property is highly advantageous in clinical practice, as it supports more transparent and interpretable dosing decisions and improves generalizability to external populations.

### 3.4 Evaluation of model‐based dose recommendation

To evaluate the mechanism‐informed ML model for individualized dose recommendation, we conducted a retrospective analysis comparing model‐based dose recommendations with standard clinical practice. The GRU model generated personalized dosing recommendations targeting the therapeutic tacrolimus C_trough_ range of 8–12 ng/mL, the target range during the first month following kidney transplantation at the University of Michigan Hospital. For comparison, dose recommendations were also generated using the PopPK model via forecast simulation. The clinically administered tacrolimus dose guided by conventional TDM was compared with the dosing range recommended by both the GRU and PopPK models. We then analysed the distribution of observed C_trough_ values measured during the first 7 days post‐transplant (excluding the first trough level), stratified by whether the actual administered dose was within the model‐recommended range.

**FIGURE 3:**
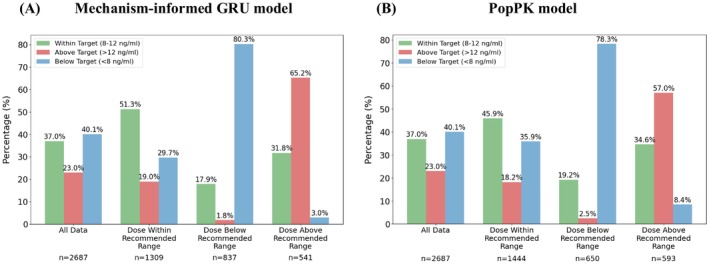
Distribution of observed tacrolimus trough concentrations stratified by whether the actual administered dose fell within the model‐recommended dosing range for (a) the mechanism‐informed GRU model and (b) the PopPK model. Observed data (excluding the first post‐transplant trough measurement) are categorized into the following groups: all data (all available through concentrations), dose within recommended range (administered dose matched the model‐recommended range), dose below recommended range and dose above recommended range. Within each group, trough concentrations are classified as below target (< 8 ng/mL, blue bars), within target (8–12 ng/mL, green bars) or above target (> 12 ng/mL, red bars).

As shown in Figure 3, overall, 37.0% of C_trough_ measurements fell within the therapeutic target range, consistent with previously reported data.^15^ When the administered dose matched the GRU model's recommendation, 51.3% of C_trough_ values were within the target range, compared to only 23.4% when the administered dose fell outside the GRU‐recommended range. In comparison, the PopPK model achieved 45.9% of target attainment when the administered doses were within its recommended range. These results highlight the improved performance of the mechanism‐informed GRU model in guiding tacrolimus dosing to achieve therapeutic targets.

**FIGURE 4:**
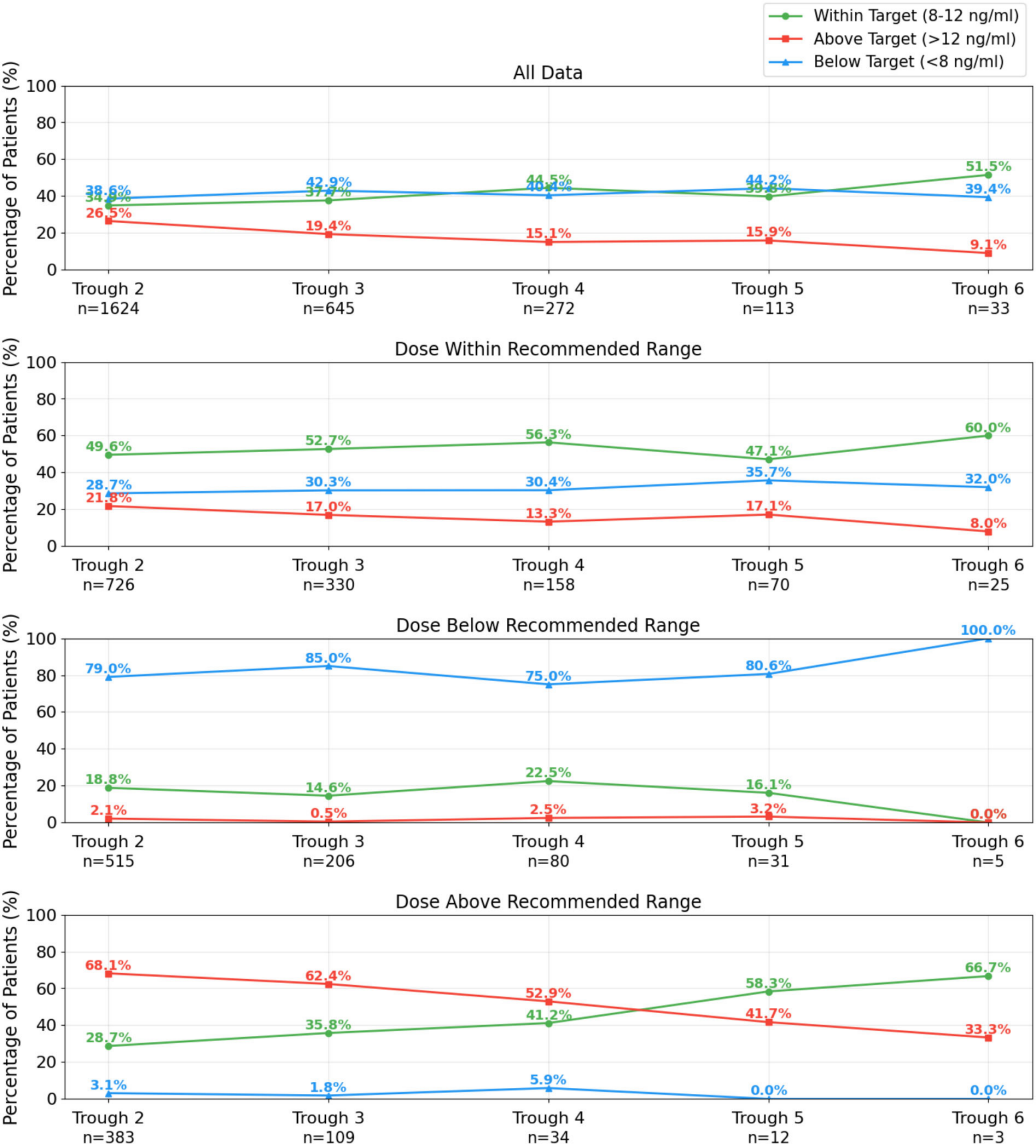
Temporal trends in the proportion of patients within, above and below the target trough concentration range over the first 7 days post‐transplant (starting from the second trough measurement). The data are stratified based on whether the actual administered dose was within the mechanism‐informed GRU model‐recommended dosing range.

Moreover, the proportion of patients achieving therapeutic target levels (excluding the first trough measurement) increased over time when the actual administered dose matched the GRU model recommendation. As shown in Figure 4, at the second trough measurement, 49.6% of patients within the model‐recommended dosing range were within the therapeutic target, increasing to 60.0% by the sixth measurement. In contrast, within the overall patient population, only 34.9% were within the target range at the second measurement, increasing to 51.5% by the sixth, consistently lower than that observed in patients receiving tacrolimus doses within the model‐recommended range. These findings suggest that our model‐recommended dosing strategy may substantially increase the likelihood of achieving and maintaining therapeutic tacrolimus levels during the early post‐transplant period, potentially improving clinical outcomes and minimizing the risks associated with under‐ and over‐exposure.

### 3.5 Feature importance analysis

**FIGURE 5:**
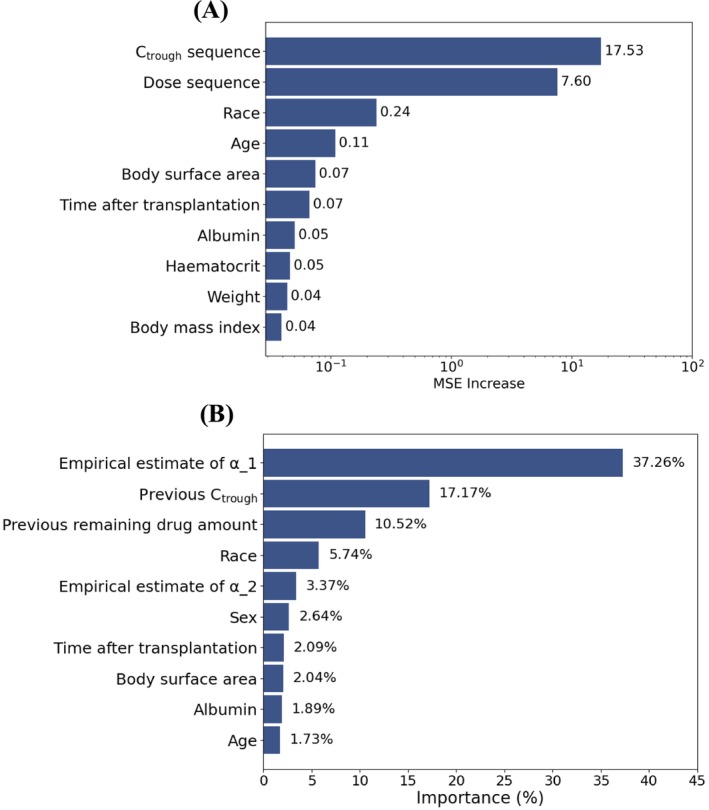
Feature importance analysis. (a) Permutation‐based feature importance in the mechanism‐informed GRU model, where importance is measured by the increase in mean squared error (MSE) after random shuffling of each feature. (b) Gain‐based feature importance in the mechanism‐informed XGBoost model, reflecting each feature's relative contribution to improving predictive performance across tree splits. Subscripts “1” and “2” indicate one‐ and two‐measurement lag, respectively. Features labeled as “previous” without subscripts refer to one‐measurement lag by default.

Feature importance analysis is presented in Figure 5. For the GRU model, the most influential predictors included the sequence of history tacrolimus C_trough_ and doses, race, age and body surface area. For the XGBoost model, the most important features were the previous empirical estimate of parameter α (37.3%), previous C_trough_ (17.2%) and the previous remaining drug amount (10.5%). Race, age and body surface area also appeared among the top 10 predictors. The prominence of race as a predictor is biologically plausible, since it may serve as a surrogate for CYP3A5 genotype in the absence of genetic data, given the higher prevalence of CYP3A5 expressors among African American individuals.^36^

### 3.6 Dose prediction software

To demonstrate the practical utility of our approach, we developed a web‐based tool based on the final mechanism‐informed GRU model to provide personalized tacrolimus dosing recommendations. Users can input patient‐specific data, including prior tacrolimus doses, corresponding C_trough_, key clinical covariates and target trough level to obtain a recommended dose for the next administration. The tool incorporates safety features such as alerts for excessive dose adjustment (i.e., exceeding 1.5 times the prior dose) and references for potential drug–drug interactions to support safer dosing decisions. The current version of this dosing tool is intended solely for research and investigational purposes and is not approved for clinical use. The web application can be accessed at https://tac-dose-adjustments.streamlit.app or via GitHub at https://github.com/huiyum/tac_dose_adjustments.

## 4 DISCUSSION

This study developed and validated mechanism‐informed ML models to predict tacrolimus C_trough_ and guide dose adjustments in kidney transplant recipients during the early post‐transplant period. Both the GRU and XGBoost models, informed by PK principles, outperformed traditional PopPK modelling, demonstrating superior and robust predictive performance in internal and external validations.

Although PopPK modelling is widely used in MIPD, its performance may be compromised in dynamic clinical scenarios involving rapidly changing physiology, such as the early post‐kidney transplant period.^20^, ^37^ In contrast, ML offers a flexible, data‐driven alternative capable of integrating high‐dimensional, time‐sensitive data and capturing complex, non‐linear patterns, making it a promising complement or substitute to traditional PopPK modelling. However, conventional ML approaches are often criticized for limited interpretability and a lack of mechanistic insight.

To address these limitations, our study employed a mechanism‐informed ML strategy rather than directly predicting tacrolimus doses or concentrations. This strategy offers several advantages. First, grounding predictions in established pharmacological principles enhances model interpretability and clinical trust. Second, decomposing the prediction task into biologically meaningful parameters allows the model to better capture inter‐individual variability. This approach preserves a linear dose–exposure relationship and reduces the instability often seen in models that directly predict drug concentrations, where small changes in dose can lead to unpredictable shifts in predictions. Third, purely data‐driven ML models, which learn statistical patterns rather than underlying mechanisms, often struggle with simulation and extrapolation tasks. While they may perform well within the range of training data, their predictions can become unreliable when applied to out‐of‐distribution data. In contrast, our mechanism‐informed ML models maintained reliable performance in external validation, despite substantial differences in patient characteristics between the training and external validation cohorts. While this variability led to an expected decrease in R^2^ during external validation, it provided a rigorous and meaningful test of model generalizability. Importantly, other metrics, such as MPE and RMSRPE, remained within clinically acceptable ranges. These results demonstrate that mechanism‐informed ML models are more robust than purely data‐driven approaches in heterogeneous external cohorts and may be better suited to support personalized tacrolimus dosing.

While the mechanism‐informed ML models outperformed the traditional PopPK model, the observed R^2^ values (~0.53) indicate moderate goodness‐of‐fit. This is expected and aligns with prior work on tacrolimus C_trough_ prediction in the early post‐transplant period, where reported R^2^ values were below 0.4.^29^ Several factors likely contribute to this limitation. First, the early post‐transplant period is marked by substantial inter‐ and intra‐individual variability due to rapidly changing physiology. Second, our models were trained exclusively on trough concentrations, which provide only single time‐point information and do not fully characterize the underlying PK profile. Third, most patients in our study cohort had only two TDM measurements within the first 7 days post‐transplant, limiting the model's ability to learn complex temporal patterns, especially for recurrent neural network architectures like GRU. Despite these challenges, the mechanism‐informed GRU model achieved a low MAE (2.02 ng/mL), minimal bias (MPE = 4.77%), and a significantly improved target attainment when administered doses aligned with model recommendations (51.3% *vs*. 23.4%, P < 0.01). These results underscore the model's potential clinical utility in supporting dosing decisions.

We also developed a web‐based tool based on the final mechanism‐informed GRU model to provide individualized tacrolimus dosing recommendations for kidney transplant recipients. This tool is intended to illustrate how the ML model could be integrated into clinical practice to support dose adjustment decisions. Clinicians can input prior doses, measured C_trough_, relevant clinical covariates and target therapeutic levels to generate a recommended dose for the next administration. While this tool is currently intended for research and investigational use only, its practical utility could be greatly enhanced through integration with electronic health record systems. Such integration would enable automated retrieval of relevant clinical data, reducing the need for manual data entry. Embedding the model into existing clinical workflows would allow dosing recommendations to be generated seamlessly in response to new TDM results, thereby improving the timeliness and accuracy of dose adjustments.

Several limitations of this work should be acknowledged. First, the dataset consisted of sparse, trough‐only concentration measurements, which limited our ability to estimate a fully time‐varying elimination rate. To accommodate this, we adopted a simplified modelling approach using an effective k_e_ at each time point. While this approach is appropriate for sparse real‐world datasets, richer sampling could improve model accuracy and support the estimation of a more physiologically realistic, time‐varying elimination profile. Moreover, reliance on C_trough_ alone does not capture the complete PK profile, and some studies have reported a relatively weak correlation between C_trough_ and overall drug exposure (AUC_0–12_), limiting its ability to fully account for inter‐ and intra‐patient variability. The 2019 IATDMCT consensus report recommends AUC‐based monitoring as a more reliable strategy during the early post‐transplant period.^9^ Developing more advanced models capable of accurately estimating AUC from sparse sampling may represent a valuable strategy for optimizing tacrolimus dosing.

Second, although the model's performance was externally validated, the retrospective study design may introduce selection bias. Third, the model was developed specifically for patients receiving the most common twice‐daily oral tacrolimus formulation, and may have limited applicability to individuals on once‐daily dosing regimens and other formulations, such as injections and oral suspensions. Additionally, the dataset was restricted to the first 7 days post‐transplant. Extending the model to support dosing decisions in the early outpatient phase is a logical next step and may enhance long‐term therapeutic management.

Future work should focus on prospective clinical trials to evaluate the impact of model‐guided dosing on clinical outcomes, including time to therapeutic range and incidence of adverse clinical events, such as rejection and nephrotoxicity. Furthermore, model‐informed initial tacrolimus dosing strategies can be integrated with this adaptive dose adjustment approach to further improve the early attainment of therapeutic targets.

## 5 CONCLUSIONS

This study developed and evaluated mechanism‐informed ML models to support individualized tacrolimus dosing during the early post‐kidney transplant period. By incorporating PK principles into GRU and XGBoost architectures, the models achieved superior predictive performance compared to traditional Bayesian PopPK and offered improved interpretability than purely data‐driven ML models. Our findings demonstrate that the mechanism‐informed ML approach holds strong potential to advance precision pharmacotherapy for kidney transplant recipients.

## AUTHOR CONTRIBUTIONS

Hui Yu conducted the study, performed data analysis and drafted the manuscript. Zihan Qin and Logan S. Smith contributed to data collection and analysis. Jeong M. Park contributed to the study's conception and offered guidance. Hao‐Jie Zhu contributed to the study's conception and design, conducted the study and provided guidance throughout the research process. All authors critically revised the manuscript and approved the final version.

## CONFLICT OF INTEREST STATEMENT

The authors declare no conflict of interest.

## CODE AVAILABILITY

The code for data processing and machine learning model development is available at: https://github.com/huiyum/tac_dose_adjustments.

## Supporting information


**Figure S1** Distribution of tacrolimus dosing and trough concentrations in the Michigan cohort. (a) Daily administered tacrolimus doses. (b) Tacrolimus trough concentration levels.
**Figure S2** Distribution of tacrolimus trough concentration entries per patient in the Michigan cohort.
**Figure S3** Tacrolimus trough concentrations over time since the first dose in the Michigan cohort. Colour‐coded by individual measurement sequence.
**Table S1.** Temporal distribution of tacrolimus TDM measurements after the initial dose in the first week post‐transplant.
**Table S2.** Model selection process for the population pharmacokinetic analysis.
**Table S3.** Parameter estimates of the population pharmacokinetic model.
**Figure S4** Goodness‐of‐fit plots for the population pharmacokinetic model. (a) Observed concentrations *vs.* population predictions; (b) Observed concentrations *vs.* individual predictions.
**Figure S5** Distribution of tacrolimus dosing and trough concentrations in the external dataset. (a) Daily administered tacrolimus doses. (b) Tacrolimus trough concentration levels.
**Figure S6** Predicted tacrolimus trough concentrations across different doses using purely data‐driven and mechanism‐informed models for a representative patient. (a) Purely data‐driven XGBoost model. (b) Mechanism‐informed XGBoost model. (c) Purely data‐driven GRU model. (d) Mechanism‐informed GRU model.

## Data Availability

The datasets are available from the corresponding author upon reasonable request.
