# Supplementary material for: Mechanism‐informed machine learning for individualized tacrolimus dose adjustment in the early post‐kidney transplant period
Source: Br J Clin Pharmacol. 2026 Jan 13;92(6):1730–43. doi: 10.1002/bcp.70448 (PMC13206353; doi:10.1002/bcp.70448)
Supplement: Supplementary file 1 — Figure S1 Distribution of tacrolimus dosing and trough concentrations in the Michigan cohort. (a) Daily administered tacrolimus doses. (b) Tacrolimus trough concentration levels. Figure S2 Distribution of tacrolimus trough concentration entries per patient in the Michigan cohort. Figure S3 Tacrolimus trough concentrations over time since the first dose in the Michigan cohort. Colour‐coded by individual measurement sequence. Table S1. Temporal distribution of tacrolimus TDM measurements after the initial dose in the first week post‐transplant. Table S2. Model selection process for the population pharmacokinetic analysis. Table S3. Parameter estimates of the population pharmacokinetic model. Figure S4 Goodness‐of‐fit plots for the population pharmacokinetic model. (a) Observed concentrations vs. population predictions; (b) Observed concentrations vs. individual predictions. Figure S5 Distribution of tacrolimus dosing and trough concentrations in the external dataset. (a) Daily administered tacrolimus doses. (b) Tacrolimus trough concentration levels. Figure S6 Predicted tacrolimus trough concentrations across different doses using purely data‐driven and mechanism‐informed models for a representative patient. (a) Purely data‐driven XGBoost model. (b) Mechanism‐informed XGBoost model. (c) Purely data‐driven GRU model. (d) Mechanism‐informed GRU model. [file BCP-92-1730-s001.pdf]

British Journal of Clinical Pharmacology

Supplementary Information

Mechanism-Informed Machine Learning for Individualized Tacrolimus Dose Adjustment in the Early Post-Kidney Transplant Period

Hui Yu[1], Zihan Qin[2], Logan Smith[2], Jeong M. Park[2], Hao-Jie Zhu[2]

[1] Department of Pharmaceutical Sciences, University of Michigan, Ann Arbor, MI, 48109, United States

[2] Department of Clinical Pharmacy, University of Michigan, Ann Arbor, MI, 48109, United States

Corresponding author: Hao-Jie Zhu, Ph.D., hjzhu@umich.edu

Department of Clinical Pharmacy, University of Michigan College of Pharmacy,

428 Church Street, Room 4565, Ann Arbor, MI 48109-1065

(a)

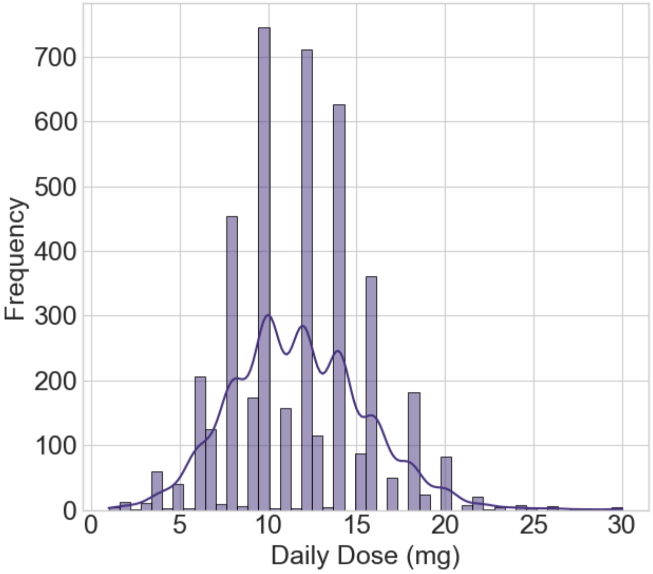

(b)

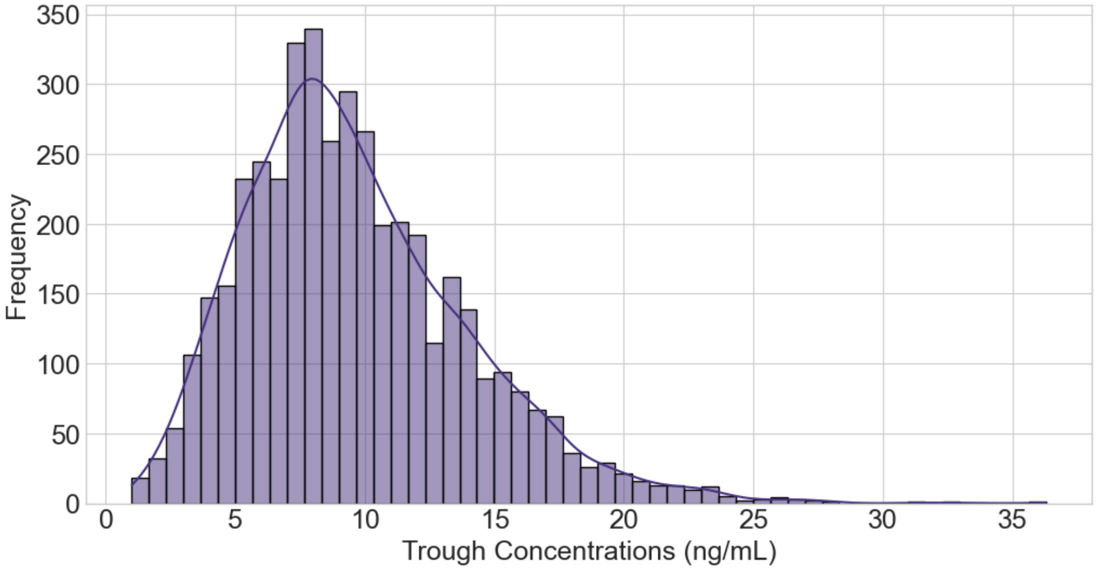

Figure S1 Distribution of tacrolimus dosing and trough concentrations in the Michigan cohort. (a) Daily administered tacrolimus doses. (b) Tacrolimus trough concentration levels.

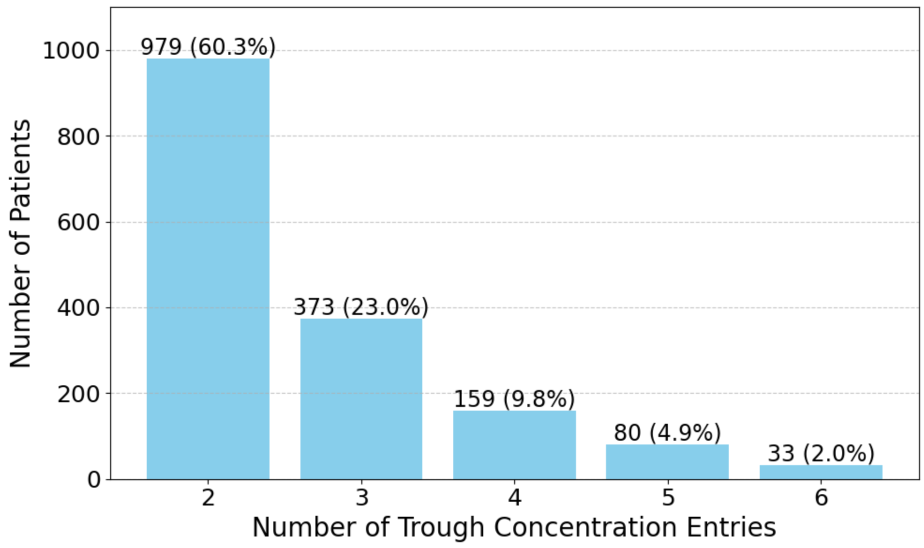

Figure S2 Distribution of tacrolimus trough concentration entries per patient in the Michigan cohort.

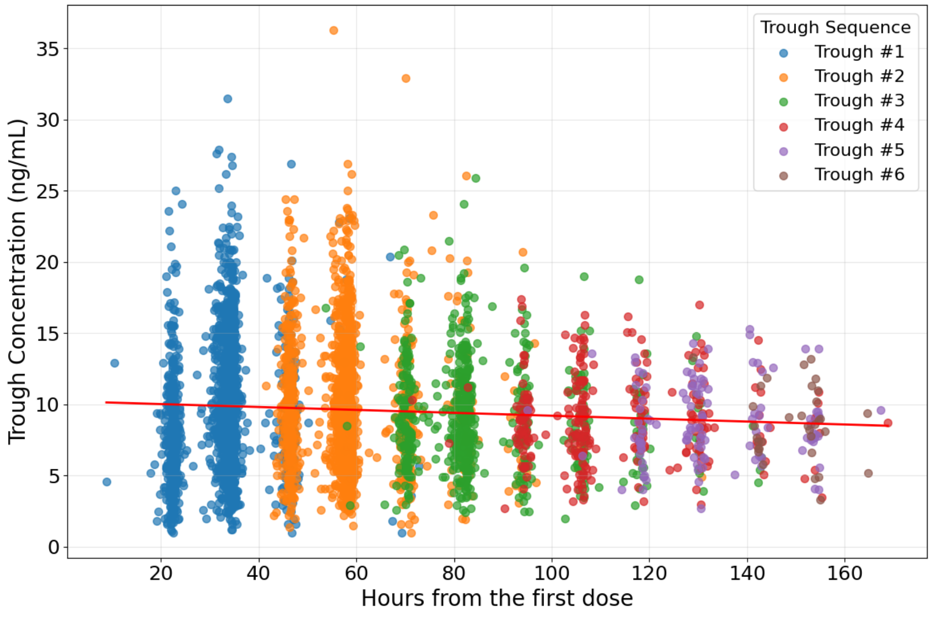

Figure S3 Tacrolimus trough concentrations over time since the first dose in the Michigan cohort. Color-coded by individual measurement sequence.

Table S1. Temporal distribution of tacrolimus TDM measurements after the initial dose in the first week post-transplant

Time Interval	Number of Observations
0–24 h	349
24–48 h	1,572
48–72 h	1,370
72–96 h	552
96–120 h	269
120–144 h	144
144–168 h	55

Table S2. Model selection process for the population pharmacokinetic analysis

Model	Description & Main characteristics	OFV	△OFV
1	Base model	23372.99	
2	Add Race on Clearance in Model 1	23222.26	-150.73
3	Add Weight on Clearance in Model 2	23138.30	-83.96
4	Add Age on Clearance in Model 3	23096.72	-41.58
5	Add Hematocrit on Clearance in Model 4	23079.84	-16.88

OFV: Objective function value, △OFV: Decrease in OFV

Table S3. Parameter estimates of the population pharmacokinetic model

Parameter	Value		Stoch. Approx.				Cond. Mode
			S.E.	R.S.E.(%)	P2.5	P97.5	Shrinkage (%)
**Fixed Effects**							
T_{lag}_pop	0.375						
k_a_pop	6.59						
CL_pop	24.3		0.434	1.78	23.5	25.2	9.1
Covariate effect on CL							
Age (years)	-0.307		0.0471	15.4	-0.399	-0.214	
Weight (kg)	0.692		0.0648	9.37	0.565	0.819	
Race (AA)	0.401		0.0333	8.30	0.336	0.466	
Hematocrit (%)	-0.394		0.0959	24.3	-0.582	-0.206	
V_1_pop	705						
Q_pop	8.54						
V_2_pop	7670						
**Standard Deviation of the Random Effects**							
	Value	C.V.(%)					
ω_{CL}	0.514	54.9	0.0117	2.27	0.491	0.537	
**Error Model Parameters**							
b	0.281		0.00391	1.39	0.274	0.289	

T_{lag}_pop: Population estimate of lag time; k_a_pop: Population estimate of absorption rate constant (1/h); CL_pop: Population estimate of clearance (L/h); V_1_pop: Population estimate of volume of central compartment (L); Q_pop: Population estimate of Intercompartmental clearance (L/h); V_2_pop: Population estimate of volume of peripheral compartment (L); AA: African American; ω_{CL}: IIV in clearance; b: The proportional error (unitless); CV: Coefficient of variation; S.E. Standard error; R.S.E. Relative standard error; P2.5, P97.5: 2.5th and 97.5th percentiles of the parameter estimate.

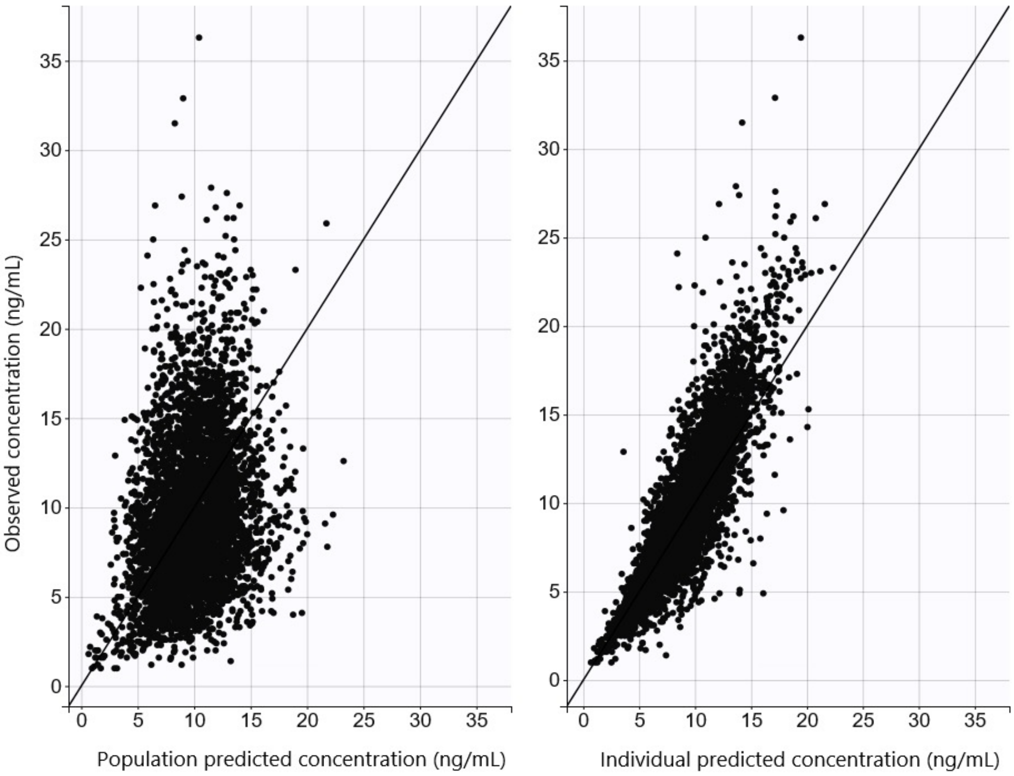

Figure S4 Goodness-of-fit plots for the population pharmacokinetic model.
(a) Observed concentrations vs. population predictions; (b) Observed concentrations vs. individual predictions

(a)

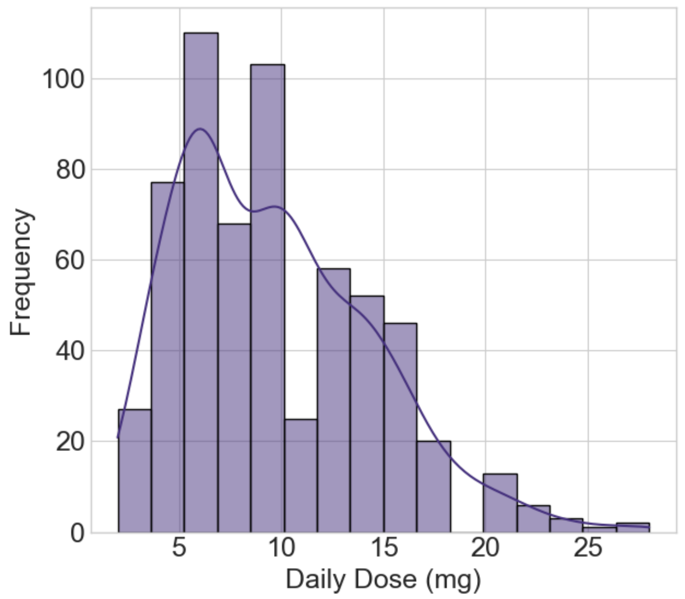

(b)

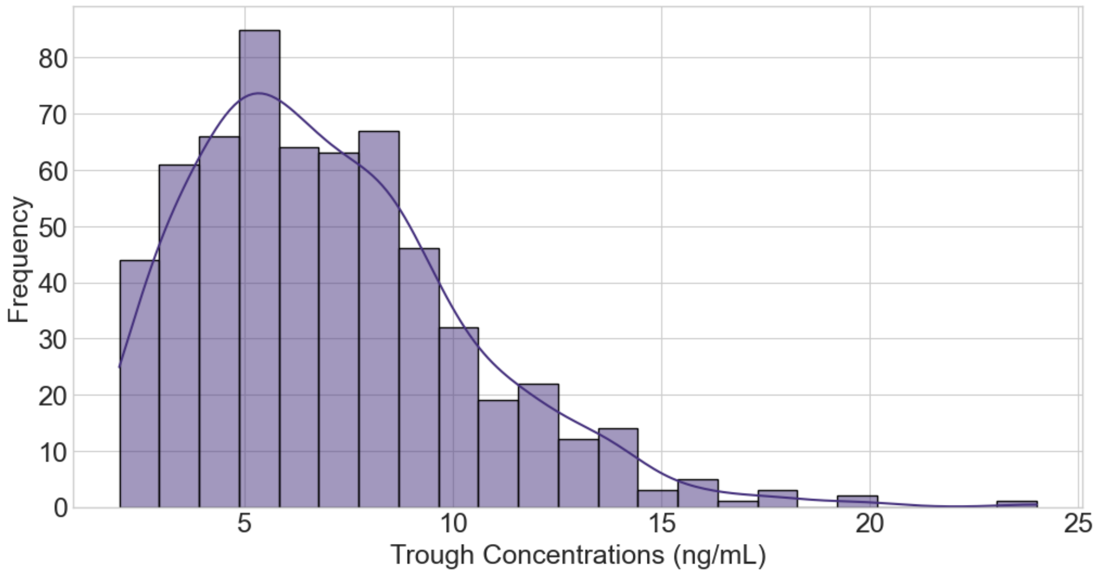

Figure S5 Distribution of tacrolimus dosing and trough concentrations in the external dataset. (a) Daily administered tacrolimus doses. (b) Tacrolimus trough concentration levels.

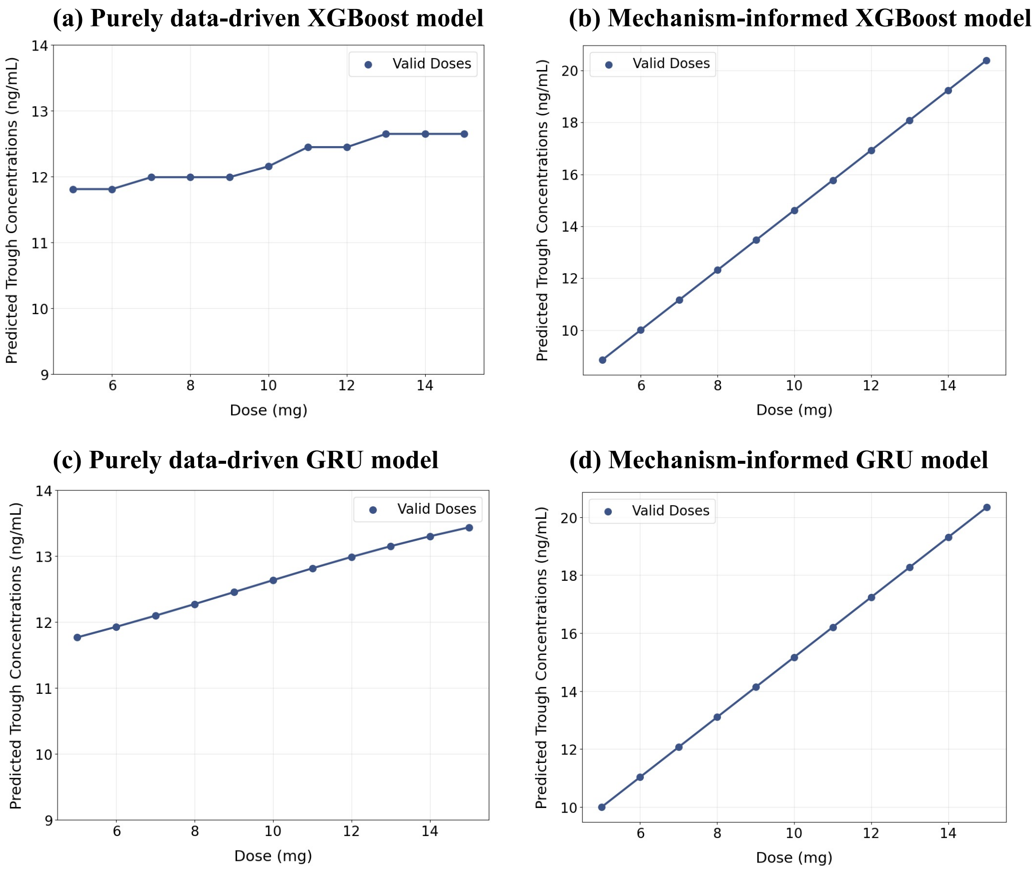

Figure S6 Predicted tacrolimus trough concentrations across different doses using purely data-driven and mechanism-informed models for a representative patient. (a) Purely data-driven XGBoost model. (b) Mechanism-informed XGBoost model. (c) Purely data-driven GRU model. (d) Mechanism-informed GRU model.
